# Barriers in screening for dementia in elderly migrants in primary care and the use of the Rowland Universal Dementia Assessment Scale. A mixed cross-sectional and qualitative study

**DOI:** 10.1080/13814788.2021.1913116

**Published:** 2021-04-30

**Authors:** Birgitte Schoenmakers, Tom Robben

**Affiliations:** Academic Centre of General Practice, Department of Public Health and Primary Care, University of Leuven, Leuven, Belgium

**Keywords:** Cognitive disorders, dementia, quality of care, primary care, migrant population

## Abstract

**Background:**

In the migrant population, there is a higher risk of dementia. However, dementia is underdiagnosed in this population due to the underuse of appropriate screening tools. The Rowland Universal Dementia Assessment Scale (RUDAS) is designed for culturally and linguistically diverse populations.

**Objectives:**

To gain insights into the primary care cognitive screening strategy for dementia in migrants and to evaluate the experiences with the RUDAS tool by general practitioners (GPs).

**Methods:**

GPs were questioned about barriers in dementia screening in the migrant population and asked to apply the RUDAS. A mixed-methods study was conducted among Flemish GPs. In an online survey, the currently used methods for screening for dementia in migrants and the barriers were investigated. In a qualitative pilot study, the user experience of the RUDAS scale was explored.

**Results:**

A total of 122/322 GPs participated (response rate 38%), 102 (83.6%) of all responding GPs mentioned language problems as the most apparent barrier. Most GPs believed the Mini Mental State Examination was inappropriate for migrants but they were unaware of an alternative. Due to lack of time and target population, only two GPs effectively applied the RUDAS. The tool was found easy to understand and less challenging in case of language problems. The main reason for not using RUDAS was the suspected time consumption.

**Conclusion:**

GPs find screening for dementia in migrant patients a challenging act, mainly due to language barriers. GPs are not confident enough with the appropriate screening strategies and tools adjusted to the needs of migrant patients. **Abbreviations:** GP: general practitioner; RUDAS: Rowland Universal Dementia Assessment Scale; MMSE: Mini Mental State Examination.

KEY MESSAGES

- GPs experience in particular language barriers and restraints from patients and family members in dementia screening in patients with a migrant background.
- GPs are not familiar with an appropriate screening tool for dementia screening in this population.
- The Rowland Universal Dementia Assessment Scale might facilitate the screening process but GPs fear the time pressure during a regular consultation.

## Introduction

Dementia is rapidly increasing worldwide and one of the most critical health challenges of the twenty-first century. The Flanders Centre of Expertise on Dementia estimated that the number of 122,000 people who had dementia in 2015 doubles by 2050. Healthcare for migrants in Western Europe is gaining attention due to the increase in migration [1].

The higher risk of dementia in the ageing migrant population as compared to the native population is, partially, due to lower educational levels [2]. Studies showed that persons not attending secondary school have a 59% higher risk of dementia [3]. Further, migrants are more susceptible to dementia-related risk factors such as depression and diabetes [4].

Multiple studies suggest that dementia is under-diagnosed in migrants [2,5]. There are language barriers, cultural barriers, lower educational levels and illiteracy, shame and stigmatisation, negative experiences and limited access to care facilities. Most healthcare professionals lack knowledge about these obstacles [6].

Another issue is the limited applicability of cognitive screening tests in migrants, due to language problems, illiteracy or low educational attainment. Language is an essential item in nearly all cognitive screening tools, including the Mini Mental State Examination (MMSE). Illiteracy leads to underperformance on measures of naming, comprehension, verbal abstraction, figure recognition and orientation. In addition, illiterate people are less familiar with tests and procedures. Therefore, low-educated or illiterate migrants unintendedly perform worse on standard dementia screening tests resulting in false-positive test results [7].

A screening tool should assess cognitive capacity independently of educational level and language. Rowland Universal Dementia Assessment Scale (RUDAS) is a validated cognitive screening tool specifically designed for culturally and linguistically diverse populations [8]. The RUDAS is described as a reliable instrument taking 10–15 min to administer and requiring minimal training.

This study’s aim was two-fold: to gain insights into the current primary care cognitive screening strategy for dementia in migrants and to evaluate the experiences with the RUDAS-tool by general practitioners (GPs).

## Methods

### Study design

The study was performed in a mixed qualitative-quantitative design.

First, the commonly used methods for screening and diagnosing dementia and the barriers were investigated in a quantitative cross-sectional study among Flemish GPs (from the 6th of December 2018 until the 8th of March 2019). An invitation with a participation link was e-mailed through the regional General Practitioner Network (total number of GPs reached = 322) to practising GPs.

Second, a 6-month qualitative pilot study was set up to test the user experience of the RUDAS. The GPs who agreed to take part in the qualitative pilot study received a personal invitation. This part of the study was conducted from April 2019 until October 2019. To initiate the qualitative part, RUDAS was explained using a Dutch-translated manual [9].

### Measurements and qualitative methods

The quantitative part inventoried methods and barriers in screening for dementia in migrant patients. The survey was constructed based upon consensus between evidence and expertise and consisted of 13 multiple-choice questions. This online survey was conducted using the web survey service of the KU Leuven (Limesurvey-KU Leuven).

In the qualitative part, GPs were asked to apply the RUDAS-instrument when patients expressed cognitive complaints or when the GP suspected cognitive decline. The RUDAS inventories in six tasks memory, visuospatial orientation, praxis, visuoconstructional drawing, judgement, memory recall and language (Box 1) [8]. After three months, GPs were contacted for a mid-term evaluation and after six months, semi-structured interviews took place by telephone.

Box 1 Test domains of the Rowland Universal Dementia Assessment Scale

**Table:** 

Cognitive domain	Question	Points
Registration	Given 4 grocery items to register (and recall later)	0
Visuospatial orientation	Left/right orientation with body parts	5
Praxis	Alternating hand movements with fist and palm	2
Visuoconstructional drawing	Copying image of a cube	3
Judgement	Safety precautions when crossing a street	4
Memory recall	Recalling the 4 grocery items from above	8
Language	Animal naming in 1 min (at least 8)	8
	Total score	30 (cut off <22)

### Study subjects

Practising GPs were recruited by e-mail through the regional GP-network. Index patients were recruited per participating GP but were not actively involved in the data collection. We defined migrants as persons born in non-western countries who migrated to Belgium at any age. The inclusion criteria for patients were: presenting with cognitive complaints or suspicion of cognitive decline. The patients signed a consent form after reading an information letter.

### Outcomes

In the quantitative part, questions addressed type and location of practice, estimated percentages of dementia patients, the proportion of patients with migration background and estimated number of migrants with dementia and questions about the cognitive screening experience (defined as barriers in cognitive screening in migrants, screening and applicability of tool in migrants). The GPs were also asked if they were familiar with RUDAS (yes/no answer option). The GPs could add comments to the following questions: barriers and used cognitive screening instruments. No patient information was collected. At the end of the survey, the participating GPs were invited to sign up for the qualitative pilot study.

In the qualitative part, we addressed the personal experience of the use of the RUDAS. GPs received a logbook to register background data of the index patient: gender, age, country of origin, years in Belgium, RUDAS test result and the GPs’ appreciation of the test result.

At the mid-term evaluation and after six months, the following items were discussed with the GPs in semi-structured interviews: general perception of the scale, practical use, positive and negative aspects, time aspect, strategies to presenting the screening tool to the patients, preparedness of the patients to participate, barriers and benefits and appreciation of the efficacy of the tool (Table 1).

### Analyses

For the survey, descriptive uni-and bivariate calculations were made. The qualitative data were analysed through thematic labelling according to the grounded theory approach [10]. Both authors performed this analysis in different rounds.

### Ethics

The study was approved by the Medical Ethical Board of the University Hospitals of Leuven under the number MP00764.

## Results

### Online survey on screening of dementia in a migrant population

The online questionnaire was completed by 122/322 GPs (38%) (Table 2). 66 (54%) of the GPs estimated the percentage of migrants among their patients at a maximum of 5%. 92 GPs (75%) confirmed that they experienced difficulties in the diagnostic process of dementia in migrants: language problems (102 GPs, 84%), not spontaneously reporting cognitive complaints (65 GPs, 53%) and impossibility to discuss the subject openly (48 GPs, 39%). 17 physicians (14%) reported difficulties other than the predefined ones: the need of hetero-anamnesis (*n* = 7), illiteracy (*n* = 2), minimisation of cognitive complaints by patients (*n* = 5) and the family being unwilling to accept investigation or external help (*n* = 3).

**Table 1.** Experiences of GPs (*n* = 122) in the diagnosis of dementia in the migrant population.

Question	*n* (%)
Difficulties in diagnostics	
Agree	42 (34.4)
Rather agree	50 (41.0)
Rather not agree	21 (17.2)
Not agree	9 (7.4)
Type of difficulties reported by GPs^a^	
Language	102 (83.6)
Subject cannot be discussed openly	48 (39.3)
Other^b^	17 (13.9)
Currently used screening tool	
None	15 (12.3)
MMSE	92 (75.4)
Clock drawing test	6 (4.9)
Other***	9 (7.4)
Currently used screening tool considered applicable for migrants	
Agree	11 (9.0)
Rather agree	13 (10.7)
Rather not agree	73 (59.8)
Not agree	25 (20.5)
RUDAS screening tool known	
Yes	1 (0.8)
No	121 (99.2)

^a^Numbers do not add up to the total number of participating general practitioners because multiple answers are possible; ^b^Other answers were combination of Mini Mental State Examination (MMSE) and clock drawing test (*n* = 6), combination of some components of MMSE and clock drawing test (*n* = 2), combination of MMSE and MOCA (Montreal Cognitive Assessment) (*n* = 1).

**Table 2.** Features of the participating GPs (*n* = 122) and their practice.

Feature	*n* (%)	Flanders *n* = 9204 (2020)
Gender, female	79 (64.8)	59% (active GPs)
Type of practice		
Solo practice	30 (24.6)	42%
Duo practice	30 (24.6)	23%
Group practice	58 (47.5)	32%
Medical centre	4 (3.3)	3%
Estimated percentage of patients with dementia/practice		
<1%	25 (20.5)	
1–2%	56 (45.9)	
2–5%	34 (27.9)	
>5%	7 (5.7)	
Estimated percentage of migrants/per practice		
<1%	28 (23.0)	
1–5%	38 (31.2)	
5–10%	24 (19.7)	
10–30%	22 (18.0)	
>30%	10 (8.20)	
Estimated number of migrants with dementia/per practice		
0–4	104 (85.3)	
5–9	13 (10.7)	
10–14	3 (2.5)	
≥15	2 (1.6)	

Ninety-two physicians (75%) used the MMSE as a standard cognitive screening tool and 15 GPs (12%) claimed not using a cognitive screening instrument. 98 GPs (80%) considered the currently used cognitive screening tools inappropriate for dementia screening in the migrant population (no reason mentioned). Only one GP was familiar with RUDAS.

### Qualitative pilot study on the use of the RUDAS tool

Of the 122 GPs participating in the online survey, 20 expressed their interest in testing RUDAS. Finally, 11 participants, including nine GPs and two nurses, signed in for the study.

At the mid-term evaluation, two participants (GPs) had used the RUDAS. Both confirmed the ease of use and had no remarks.

At the semi-structured telephone interviews after 6 months, participants mentioned that they had not been able to use RUDAS after the mid-term evaluation. Therefore, the study resulted in only three completed RUDAS forms by in total two GPs. The time needed to conduct RUDAS was 10–15 min. One GP regarded the RUDAS-tool as a less known but promising tool. In two cases, a family member interacted as an interpreter. Both GPs noticed that the test required fewer language skills and was easy to understand. Both GPs also stated that the test required a certain level of education and that some words were uncommon in some cultures (e.g. ‘cooking oil’ apparently is not a common term in Moroccan and Turkish cultures). Finally, the test appeared too time-consuming.

The nine other participants who had not used the RUDAS during the 6-month study period gave the following reasons: no eligible patients (*n* = 9). One GP expected the tool to be too time-consuming. The participating nurses did not have the opportunity to apply the RUDAS since no eligible patients were referred by the GPs.

## Discussion

### Main findings

Most GPs experienced difficulties in the diagnostic process of dementia in the migrant group. The most common barrier in this process was related to language issues. Only one GP was familiar with the RUDAS. All other GPs used the commonly available screening instruments and reported the lack of adequate assessment tools in the migrant population. The RUDAS was used in only three cases and was found easy to understand and less challenging in language problems. The main reasons for not applying the RUDAS were the shortage of eligible patients and a perceived lack of time during a 15 min patient encounter.

### Strengths and weakness

The major weakness of the study is the low participation rate in the qualitative survey. GPs dropped out because of the lack of patients and the fear that the RUDAS’ application would put the consultation under time pressure. Although, a lack of eligible patients might be inherent to the health beliefs and literacy of the target population. Dementia is not a commonly used term among migrants and they prefer using forgetfulness as this term is less loaded and considered inherent to ageing. Migrant patients and their families might, therefore, doubt the usefulness of dementia screening [11]. Second, the perceived lack of time to apply the RUDAS contrasts with the actual time needed for the administration of a regular cognitive screening instrument, which also takes 10–15 min [12].

To our knowledge, our survey is the first in Belgium that observed GPs’ experiences with cognitive screening in migrants. The survey resulted in 122 completed questionnaires, one of the largest samples on this topic in the international literature. Despite the small sample size, the contribution rate of GPs’ characteristics represents the Flemish GP population (Table 2) (https://www.inami.fgov.be/nl/statistieken/Paginas/default.aspx). Second, in contrast to our survey, the GPs participating in the study by Vissenberg et al. were all frequently working with migrants [11]. However, using experience as an inclusion criterion, selection bias heads up.

### Interpretation of results

The estimated percentage of patients who have dementia in a GP practice is about 1–2% of which less than four patients have a migrant background. This estimated prevalence, of dementia, is in line with the Flemish statistics [13]. Older migrants are a minor population group in Flanders and the migrant population is significantly younger than the native Belgian population (https://statbel.fgov.be/en). Most participating GPs experienced difficulties in the diagnostic process of dementia in migrants, consistent with the findings in a recent study [11]. In our study, language issues were the most frequently mentioned problems. Some GPs in our study also faced the unwillingness of patients and families to participate in the diagnostic process and during follow-up. In similar research, GPs indicated that they could not treat migrants with dementia as they wished [6]. Above, GPs perceive that migrants will not consent to perform cognitive screening tests due to religious or cultural beliefs [14].

Most GPs in our study used the MMSE to screen for cognitive decline and they reported the lack of adequate assessment tools to use in the migrant population. Most of the GPs did not know the RUDAS. From studies in dementia clinics, we have learned that under-diagnosis is a major problem and likely due to the use of an inappropriate screening instrument [15].

In our study, most GPs stated that they could not perform brief cognitive screening tests in migrants because of communication problems or illiteracy. Due to low educational levels and poor health literacy, migrant patients’ knowledge about dementia often is too low to contribute to the assessment process [6,11].

In the qualitative pilot study, we only collected three fully completed RUDAS forms. The drop out of GPs in the qualitative study was mainly due to a shortage of eligible patients and a perceived lack of time during a 15 min patient encounter. The administration of a cognitive screening instrument takes 10–15 min (e.g. Mini Mental State Examination, Clock Drawing Test) [12]. This observation contrasts with the finding that most of the participating GPs claimed using MMSE, which takes as long as using RUDAS. This contradiction might be explained by the fact that GPs depart from a clinical assessment and only proceed to a formal cognitive test in case of doubt [12]. Above, GPs tend to refer patients for further neuropsychological evaluation [16]. Nevertheless, guidelines recommend applying a cognitive screening tool before referring patients and addressing the diagnostic process in multiple steps [17]. A cognitive screening test is the second step, in a separate consultation. Migrants are often lost to follow up at that point [18,19]. The last reason for not using a cognitive screening tool is the reporting of GPs that they are not familiar with tools other than the MMSE and the Clock Drawing Test. This finding is in agreement with other research [20].

## Conclusion

GPs find screening for dementia in migrant patients a challenging operation. In particular, language barriers lead to the under-diagnosis of dementia in this particular population. GPs are not confident enough with the appropriate screening strategies and fear a time-consuming process.

The inventory of barriers in this screening process might add to the support and education of GPs in contact with a migrant patient presenting with cognitive decline. The use of appropriate screening tools should be brought to the attention of GPs and supported to implement in daily practice.

## Data Availability

The complete dataset is available on request and will be sent as a link to a google-drive directory
